# Kaposi’s Sarcoma Herpesvirus Genome Persistence

**DOI:** 10.3389/fmicb.2016.01149

**Published:** 2016-08-12

**Authors:** Franceline Juillard, Min Tan, Shijun Li, Kenneth M. Kaye

**Affiliations:** Departments of Medicine, Brigham and Women’s Hospital, Harvard Medical SchoolBoston, MA, USA

**Keywords:** KSHV, latency-associated nuclear antigen, chromosome, DNA binding, viral persistence

## Abstract

Kaposi’s sarcoma-associated herpesvirus (KSHV) has an etiologic role in Kaposi’s sarcoma, primary effusion lymphoma, and multicentric Castleman’s disease. These diseases are most common in immunocompromised individuals, especially those with AIDS. Similar to all herpesviruses, KSHV infection is lifelong. KSHV infection in tumor cells is primarily latent, with only a small subset of cells undergoing lytic infection. During latency, the KSHV genome persists as a multiple copy, extrachromosomal episome in the nucleus. In order to persist in proliferating tumor cells, the viral genome replicates once per cell cycle and then segregates to daughter cell nuclei. KSHV only expresses several genes during latent infection. Prominent among these genes, is the latency-associated nuclear antigen (LANA). LANA is responsible for KSHV genome persistence and also exerts transcriptional regulatory effects. LANA mediates KSHV DNA replication and in addition, is responsible for segregation of replicated genomes to daughter nuclei. LANA serves as a molecular tether, bridging the viral genome to mitotic chromosomes to ensure that KSHV DNA reaches progeny nuclei. N-terminal LANA attaches to mitotic chromosomes by binding histones H2A/H2B at the surface of the nucleosome. C-terminal LANA binds specific KSHV DNA sequence and also has a role in chromosome attachment. In addition to the essential roles of N- and C-terminal LANA in genome persistence, internal LANA sequence is also critical for efficient episome maintenance. LANA’s role as an essential mediator of virus persistence makes it an attractive target for inhibition in order to prevent or treat KSHV infection and disease.

## Introduction

Kaposi’s sarcoma-associated herpesvirus (KSHV) (or human herpesvirus 8) is a large, enveloped, double-stranded DNA virus. KSHV is the only human gamma-2 herpesvirus, belonging to the Rhadinovirus genus. The KSHV genome is ~165 kb, and encodes nearly 100 genes. Similar to all herpesviruses, after infection, KSHV persists for the lifetime of its host. KSHV persists by latently infecting cells. During latent infection, the viral genome exists as a multi copy, extrachromosomal, circular episome (plasmid). During latent infection, only a small subset of viral genes is expressed. These genes include the latency-associated nuclear antigen (LANA), v-FLIP, and v-Cyclin (Chang et al., 1996; Kedes et al., 1997; Kellam et al., 1997; Rainbow et al., 1997; Dittmer et al., 1998). Low levels of K1 transcript are also detected in latent infection, and v-IL6 (K2) is expressed at low levels during latency in certain cell types (Chandriani and Ganem, 2010). vIRF3 (LANA2) is expressed during latent infection, but only in B cells (Rivas et al. 2001). The KSHV K12 locus encodes viral miRNAs which are expressed in latency (Samols et al., 2005), and this locus also encodes kaposins A, B, and C, of which the kaposin B protein is the predominant product and most easily detected during lytic infection (Sadler et al., 1999).

## LANA Mediates KSHV Genome Persistence

**FIGURE 1:**
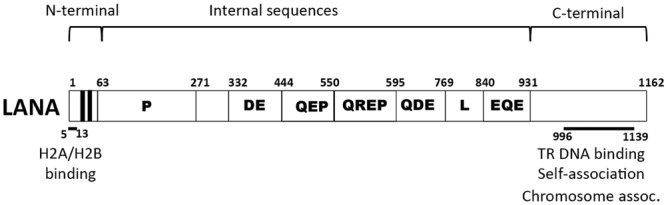
**Schematic diagram of Kaposi’s sarcoma-associated herpesvirus (KSHV) latency-associated nuclear antigen (LANA).** Indicated are the proline-rich region (P), and the repetitive regions (DE, QEP, QREP, QDE, L, EQE). The black areas indicate the bipartite N-terminal nuclear localization signal (NLS) within amino acids 24 to 30 and 41 to 47. Amino acids 5 to 13 mediate chromosome association through interaction with histones H2A/H2B. Amino acids 996 to 1139 contain TR DNA binding, self-association, and chromosome association functions.

**FIGURE 2:**
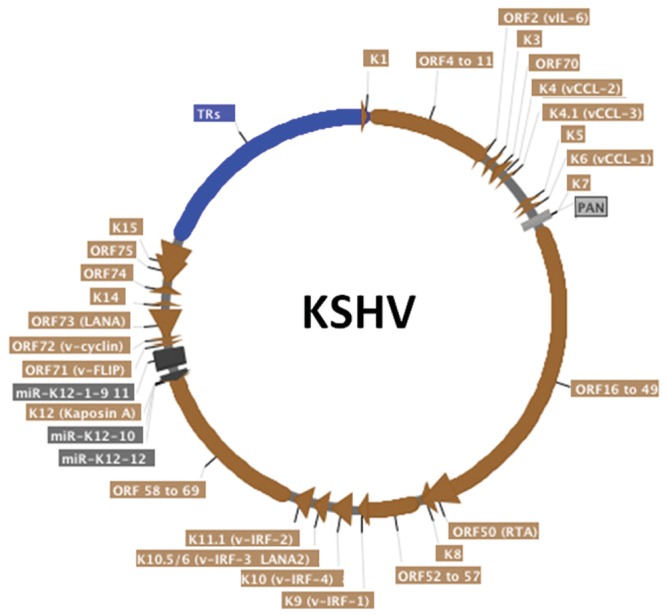
**Schematic representation of the circularized KSHV genome.** The terminal repeats (TRs), in blue, comprise around 20% of the genome. KSHV open reading frames (ORF) are in brown, miRNAs are in dark gray and non-coding long RNA PAN is in light gray. Alternative protein names are written in parenthesis. Arrows indicate transcription direction. Although KSHV persistence is the focus of this article, most KSHV genes are shown. This map was generated from the HHV-8 complete genome sequence (Genbank accession AF148805) strain GK18 using SerialCloner software.

Latency-associated nuclear antigen is an 1162 amino acid protein encoded by KSHV open reading frame (ORF) 73 (**Figure 1**) and is the viral protein responsible for virus persistence. LANA acts on KSHV terminal repeat (TR) sequence to mediate episome persistence. The viral TRs are 801 bp repeated sequences that are highly GC-rich (~80%) and comprise ~ 20% of the genome (**Figure 2**). Upon initial infection, the linear KSHV genome circularizes by fusing at the TR elements, located at each end of the genome. LANA is necessary and sufficient to mediate episome persistence of TR associated DNA in the absence of other virus genes (Ballestas et al., 1999; Ballestas and Kaye, 2001; Garber et al., 2001; Fejer et al., 2003; Grundhoff and Ganem, 2003, 2004; Skalsky et al., 2007). Knockdown of LANA using siRNA leads to loss of episomes, while deletion of LANA from a bacterial artificial chromosome (BAC) containing the viral genome results in loss of the ability of virus to persist in an episomal state (Zhang et al., 2007; Li et al., 2008). Similarly, knockout or mutating MHV-68 ORF73, the murine LANA homolog, from murine γ-herpesvirus 68 abolishes the ability of virus to efficiently establish latent infection (Fowler et al., 2003; Moorman et al., 2003; Forrest et al., 2007; Paden et al., 2010; Habison et al., 2012; Correia et al., 2013).

There are two key components to episome persistence. First, episomal DNA must replicate with each cell division. Second, episomes must segregate to progeny nuclei following mitosis to avoid destruction in the cytoplasm. LANA is responsible for both of these functions. LANA mediates episomal DNA replication. LANA also serves to segregate KSHV episomes to daughter nuclei. LANA accomplishes this segregation by acting as a molecular tether for viral episomes, bridging virus DNA to host cell mitotic chromosomes.

This review focuses on current understanding of LANA’s mediation of genome persistence through tethering KSHV DNA to mitotic chromosomes to effect distribution of episomes to daughter nuclei. LANA’s role in KSHV DNA replication is addressed separately in another chapter.

### LANA Binds Chromosomes to Mediate Viral Genome Persistence

#### LANA Tethers the KSHV Genome to Host Cell Chromosomes

Using simultaneous immune fluorescence to detect LANA and fluorescent *in situ* hybridization to detect KSHV DNA, LANA was shown to colocalize with KSHV episomes along metaphase chromosomes in KSHV latently infected cells (Ballestas et al., 1999; Cotter and Robertson, 1999). This finding suggested LANA had a role in KSHV episome persistence analogous to EBNA1 of Epstein-Barr virus (EBV) (Reedman and Klein, 1973; Grogan et al., 1983; Yates et al., 1984; Harris et al., 1985), and in fact, LANA expressing cells were shown to allow persistence of plasmids containing KSHV TR DNA (Ballestas et al., 1999; Ballestas and Kaye, 2001). This work led to a model in which LANA bridges KSHV DNA to chromosomes during mitosis through concomitantly binding to the *cis*-acting TRs sequence in the KSHV genome and to mitotic chromosomes. Therefore, LANA tethers KSHV episomes to mitotic chromosomes during cell division to ensure segregation of the viral genome to the nuclei of daughter cells.

#### N- and C-terminal LANA Independently Bind Mitotic Chromosomes

Both N- and C-terminal regions of LANA are involved in mitotic chromosome binding. N-terminal LANA residues 5–22 were originally reported by Piolot et al. (2001) to target chromatin during interphase and mitosis. N-terminal LANA also encodes a nuclear localization signal, which is bipartite, located within amino acids 24 to 30 and 41 to 47. Cherezova et al. (2011) consistent with its role in tethering KSHV DNA to mitotic chromosomes, the LANA N-terminal chromosome association region was later shown to be essential for LANA mediated episome persistence. Of interest, the N-terminal region was also found to be critical for efficient LANA mediated DNA replication, despite having no role in binding KSHV DNA (Barbera et al., 2004; Lim et al., 2004). In addition, the N-terminal LANA chromosome binding region also has effects on LANA transcriptional regulation (Wong et al., 2004). Other than microscopy, biochemical evidence also supports N- and C-LANA interactions with chromatin (Viejo-Borbolla et al., 2003; Wong et al., 2004).

**FIGURE 3:**
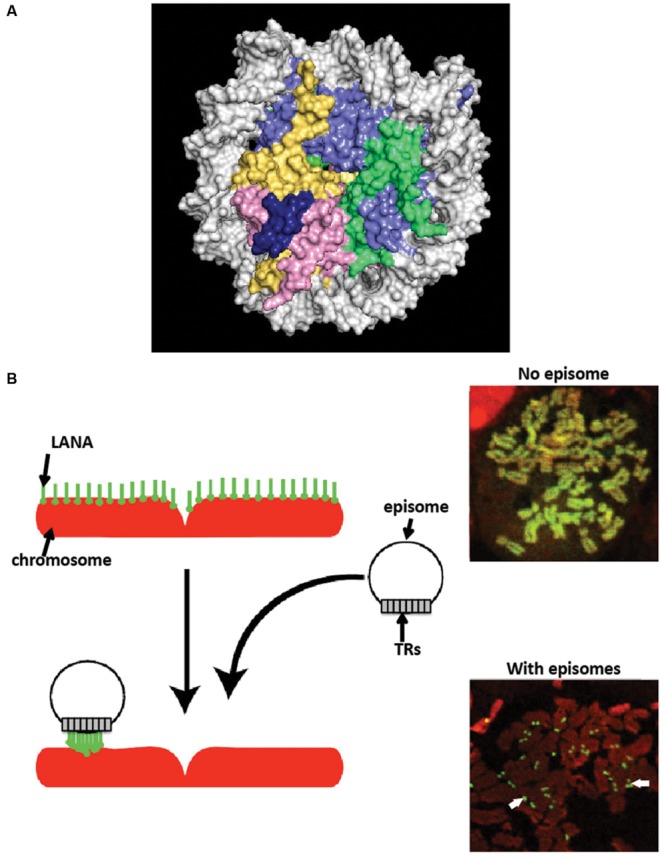
**Latency-associated nuclear antigen associates with chromosomes. (A)** X-ray crystal structure of N-terminal LANA complexed with the nucleosome. A space-filling representation is shown. (histone H2A, yellow; H2B, red; H3 light blue; H4, green; LANA, dark blue; DNA, silver). From PDB 1zla (Barbera et al., 2006a). **(B)** LANA associates with cellular chromosomes in a broad distribution in the absence of episomes, and concentrates to dots on chromosomes at sites of episomes. Cells expressing LANA were arrested in metaphase with colcemid. LANA (green) was detected with antibody directed against LANA. DNA was counterstained with propidium iodide (red) (overlay of red and green generates yellow.) White arrows indicate LANA dots at sites of KSHV episomes.

N-terminal LANA binds directly to the surface of the nucleosome to attach to chromosomes. X-ray crystal structure of N-terminal LANA complexed with a nucleosome core particle showed that the first 23 amino acids of LANA forms a hairpin that binds exclusively to the acidic patch at the interface of histones H2A and H2B (Barbera et al., 2006a) (**Figure 3A**). The nucleosome binding property of N-terminal LANA determines its broad distribution across mitotic chromosomes in the absence of episomal DNA (Piolot et al., 2001; Barbera et al., 2004, 2006a; Wong et al., 2004; Chodaparambil et al., 2007). Whether or not histone epigenetic modifications exert a modulatory role on N-terminal LANA binding to the nucleosome surface is not currently known.

C-terminal LANA contains an independent chromosome binding domain, which consists of amino acids 996–1139 (Krithivas et al., 2002; Kelley-Clarke et al., 2007a, 2009). The LANA C-terminal domain is conserved among the LANA orthologs from different gamma-2 herpesviruses, including ORF73s from Retroperitoneal fibromatosis herpesvirus (RFHV) (Burnside et al., 2006), Rhesus rhadinovirus (RRV), Herpesvirus saimiri (HVS) (Calderwood et al., 2004), Herpesvirus ateles (HVA), and murine gammaherpesivrus 68 (MHV68) (Grundhoff and Ganem, 2003; Paden et al., 2012).

C-terminal LANA binds chromatin in a unique pattern in the absence of episomal DNA, concentrating to punctate foci at pericentromeric and peri-telomeric regions of a subset of mitotic chromosomes (Krithivas et al., 2002; Kelley-Clarke et al., 2007a). This pattern contrasts sharply with the diffuse chromosome distribution of N-terminal LANA. Self-association of LANA C-terminal region is required for LANA C-terminal chromosome binding (Komatsu et al., 2004; Kelley-Clarke et al., 2009). Of other episome maintenance proteins, EBV EBNA1 does not localize to pericentromeric regions of sister chromatids during mitosis (Szekely et al., 1999; Kanda et al., 2007; Nanbo et al., 2007). The binding pattern of human papillomavirus (HPV) E2 episome maintenance protein to chromosomes can be pericentromeric or not, depending on the virus type (Oliveira et al., 2006).

#### LANA Broadly Associates with Chromatin in the Absence of Episomes, but Concentrates to Dots on Chromosomes at Sites of Episomal DNA

In the absence of KSHV episomes, full length LANA broadly distributes on mitotic chromosomes (Ballestas et al., 1999; Piolot et al., 2001). However, LANA’s distribution is uneven, and not diffuse, when expressed at physiological levels (Kelley-Clarke et al., 2009). Since N-terminal LANA diffusely paints mitotic chromosomes through binding to histones H2A/H2B, and since C-terminal LANA concentrates to pericentromeric and peri-telomeric regions of mitotic chromosomes (as described above), the result of combining these two localization targeting mechanisms in full length LANA effects broad distribution over mitotic chromosomes, but with more intense staining at peri-telomeric and pericentromeric regions.

In stark contrast to its broad chromosomal distribution in the absence of episomes, LANA concentrates to dots in the presence of KSHV episomal DNA (**Figure 3B**), both in interphase and along mitotic chromosomes (Ballestas et al., 1999; Cotter and Robertson, 1999; Sakakibara et al., 2004). The concentration to dots occurs at the sites of KSHV episomal genomes, as shown by simultaneous immune fluorescent detection of LANA and fluorescent *in situ* hybridization with KSHV DNA (Ballestas et al., 1999; Cotter and Robertson, 1999). The strong concentration of LANA to dots at sites of episomal DNA is likely a result of the higher affinity that C-terminal LANA has for its DNA binding site in TR DNA [Kd of binding to LANA adjacent binding sites 1 and 2 ~13.7 nM (Garber et al., 2002; Ponnusamy et al., 2015)] compared to a lower affinity for N-terminal LANA binding to the nucleosome [Kd ~184 nM (Beauchemin et al., 2014)]. Further, each KSHV genome contains ~40 TR copies, and each TR contains three adjacent LANA binding sites (Garber et al., 2002; Hellert et al., 2015). Therefore, each KSHV genome contains ~120 LANA binding sites within its TR elements, to which a LANA dimer binds at each site, resulting in ~240 LANA molecules binding to TR DNA per KSHV genome. LANA bound at TR DNA and simultaneously binding to nucleosomes within mitotic chromosomes results in tethering of the viral genome to mitotic chromosomes. What is less clear, however, is whether or not a subset of LANA molecules bound at TR DNA bind nucleosomes within the KSHV episome; such binding would not result in tethering to mitotic chromosomes but instead would result in LANA doubly bound to the episome: through direct TR DNA binding and also through nucleosomal attachment. If such binding occurs, it would potentially compete with binding to chromosomes, and perhaps could serve as a regulatory mechanism.

#### N-terminal LANA Is the Dominant Chromosome Attachment Region

Although both N- and C-terminal LANA contain independent chromosome binding regions, N-terminal LANA appears to be the primary effector. Alanine substitution of key chromosome attachment residues in N-terminal LANA abolished LANA’s chromosome association and its ability to mediate episome persistence (Barbera et al., 2004). In contrast, alanine substitutions that dramatically impair C-terminal LANA’s ability to bind mitotic chromosomes did not reduce full length LANA’s association with chromosomes or its ability to mediate episome persistence (Kelley-Clarke et al., 2009). It is important to note, however, that these experiments could not use LANA that was completely abolished for C-terminal chromosome binding. Such null chromosome binding mutations also impaired other critical C-terminal LANA functions, such as DNA binding. Therefore, it remains possible that N-terminal LANA may have “rescued” the impaired (but not abolished) C-terminal LANA chromosome binding, possibly through a cooperative effect. In fact, when N-terminal LANA was mutated so as to reduce (but not abolish) N-terminal LANA chromosome association, the C-terminal chromosome binding mutations resulted both in a reduction of full length LANA binding to mitotic chromosomes and also in a reduction of LANA’s ability to mediate episome persistence (Kelley-Clarke et al., 2009).

Altogether, these data suggest that N-terminal LANA is the dominant effector for chromosome binding, and that C-terminal LANA exerts an auxiliary role. It is tempting to consider that C-terminal LANA was the original chromosome tether, and that N-terminal LANA evolved subsequently as a more efficient alternative. Consistent with this possibility, C-terminal LANA is the most highly conserved region among LANA orthologs.

#### LANA, Including N-terminal LANA, Undergoes Post Translational Modifications

Latency-associated nuclear antigen has been described to undergo several types of post translational modifications. LANA is a phosphoprotein and is the target of several host cell kinases. Pim-1 and Pim-3 phosphorylate LANA serine residues 205 and 206 to allow lytic reactivation (Bajaj et al., 2006; Cheng et al., 2009). RSK1 and ERK1/2 interact with LANA along with GSK-3 and phosphorylate LANA residues located within 246–258 (Fujimuro et al., 2005; Liu et al., 2007). Notably, RSK3 phosphorylates LANA serine 13 and threonine 14, within the N-terminal chromosome binding region. RSK small molecule inhibition decreased LANA binding to histone H2B as well as LANA protein levels. Treatment of KSHV infected PEL cells with the RSK inhibitor resulted in growth cessation and a decrease in viable cell numbers. In contrast, treatment of uninfected BJAB B cell lymphoma cells resulted in growth cessation, but there was not a decrease in the number of viable cells. These findings show that inhibition of RSKs can impact KSHV persistence (Woodard et al., 2012). DNA-PK was also shown to phosphorylate LANA in at least two sites, one located between amino acids 31–52, and a second between amino acids 91–340, although the specific residues were not identified. The phosphorylation between residues 31–52 may have reduced LANA mediated DNA replication (Cha et al., 2010). C-terminal LANA is also phosphorylated at serine and threonine residues between amino acids 951–1107, and this is mediated by a kinase recruited by RING3 (Brd2) (Platt et al., 1999).

In addition to phosphorylation, N-terminal LANA arginine residue 20, located near the chromosome attachment region, was shown to be methylated by protein arginine methyltransferase 1 and to have a role in antagonizing viral lytic reactivation (Campbell et al., 2012). LANA is also acetylated, and sodium butyrate (a histone deacetylase inhibitor) treatment, reduced LANA’s ability to co immunoprecipitate with histones H2A/H2B (Lu et al., 2006). LANA is also a target of poly (ADP-ribose) polymerase 1 (PARP-1) and is poly (ADP-ribosyl) ated (Ohsaki et al., 2004). The sites of acetylation or poly (ADP-ribosyl) ation within LANA are not currently known. In addition, C-terminal LANA is SUMOylated at lysine 1140 (Cai et al., 2013).

### Cellular Proteins Associated with LANA at the Chromosome

In addition to histones, LANA interacts with other chromosome associated proteins. Although N-terminal LANA binding to histones H2A/H2B is likely the primary chromosome attachment mechanism, other cell proteins may modulate this interaction. LANA N- and C-terminal regions both interact with the methyl CpG binding protein 2 (MeCP2), which binds methylated CpG dinucleotides (Krithivas et al., 2002; Matsumura et al., 2010). MeCP2 was initially proposed to mediate N-terminal LANA chromosome association based on the inability of LANA to associate with murine chromosomes in the absence of human MeCP2, although other work demonstrated LANA associates with native murine chromosomes (Krithivas et al., 2002; Barbera et al., 2006b; Kelley-Clarke et al., 2007a). The interaction between LANA and MeCP2 may confer LANA with the potential to regulate cellular gene expression in latently infected cells (Matsumura et al., 2010). Of note, MeCP2 was shown to be important for herpesvirus saimiri LANA episome persistence (Griffiths and Whitehouse, 2007). LANA also interacts with the DEK protein, which associates with chromatin. The DEK-interacting region was mapped to LANA amino acids 986 to 1043 and DEK was proposed to mediate C-terminal LANA attachment to chromosomes (Krithivas et al., 2002), although unlike C-terminal LANA’s concentration to pericentromeres and peri-telomeres, DEK diffusely paints mitotic chromosomes (Krithivas et al., 2002; Kelley-Clarke et al., 2007a). Full length LANA was shown to interact with histone H1 (Cotter and Robertson, 1999), and a chimeric LANA with the N-terminal chromosome association region replaced by histone H1 protein was functional for episomal maintenance (Shinohara et al., 2002). Results differ as to whether histone H1 can interact with N-terminal LANA (Barbera et al., 2006a; Verma et al., 2013).

KSHV LANA also interacts with BET (Bromodomain and Extra Terminal domain) proteins (Platt et al., 1999; Ottinger et al., 2006). The BET protein BRD4 binding sites on LANA have been mapped to LANA amino acids 475 to 777 and C-terminal amino acids 982 to 1162 (Viejo-Borbolla et al., 2005; You et al., 2006). BET proteins such as BRD2 and BRD4 have two bromodomains and an extraterminal (ET) domain. BET proteins interact with C-terminal LANA through the BET protein ET domain and also through a conserved serine rich region downstream of the ET domain (Hellert et al., 2013). BET protein bromodomains bind acetylated tails of histones H3 and H4 and may have a role in LANA’s transcriptional properties (Viejo-Borbolla et al., 2005; Ottinger et al., 2006; You et al., 2006). Notably, bovine papillomavirus E2, which is an episomal maintenance protein, attaches to chromosomes through the bromodomain of BRD4 (You et al., 2004).

Nuclear Mitotic Apparatus (NuMA), a protein interacting with microtubule dynein/dynactin during mitosis, binds C-terminal LANA and associates with LANA during interphase and at the end of telophase. Repressing NuMA function led to a reduction in episomal maintenance (Si et al., 2008). LANA also associates with the kinetochore proteins CENPF and BUB1. Both N- and C-terminal LANA are involved in CENPF and BUB1 association and the interaction between CENPF and LANA leads to colocalization of LANA with these kinetochore proteins in a subset of ~50% of mitotic chromosomes in KSHV infected PEL cells (Xiao et al., 2010). Bub1, but not CENPF depletion with RNA interference led to a reduction in KSHV episome copy number in PEL cells (Xiao et al., 2010). LANA also targets BUB1 for degradation which can lead to chromosomal instability (Sun Z. et al., 2014). PARP-1 binds KSHV TR DNA. PARP-1 activity may control viral copy number in infected cells. Treatment of BC3 PEL cells with hydroxyurea, a compound that elevates PARP activity, decreased KSHV copy number and treatment with niacinamide and 3-aminobenzamide, which decrease PARP activity, increased KSHV copy numbers (Ohsaki et al., 2004). The DNA-PK/Ku complex associates with N-terminal LANA, and phosphorylates LANA. Overexpression of the catalytic subunit Ku70, but not Ku86, impaired LANA replication of a TR-containing plasmid in 293T cells. This suggests that DNA-PK/Ku may negatively regulate KSHV latent replication (Cha et al., 2010). LANA localizes to heterochromatin and interacts with heterochromatin protein 1 (HP1), which is a chromodomain containing protein, and SUV39H1, a histone methyltransferase, at chromosomes (Szekely et al., 1999; Lim et al., 2003; Sakakibara et al., 2004). Histone H2AX also interacts with LANA and its phosphorylated form, gamma H2AX colocalized with a subset of LANA dots. Knockdown of H2AX decreased KSHV episome copy number (Jha et al., 2013). HMGA1 or HMGB1 also interact with LANA and chromosomes (Shamay et al., 2012).

### LANA Binds Viral TR DNA through Its C-terminal Domain to Mediate Viral Genome Persistence

#### LANA Binds Specific Sequence in the KSHV TR Element

**FIGURE 4:**
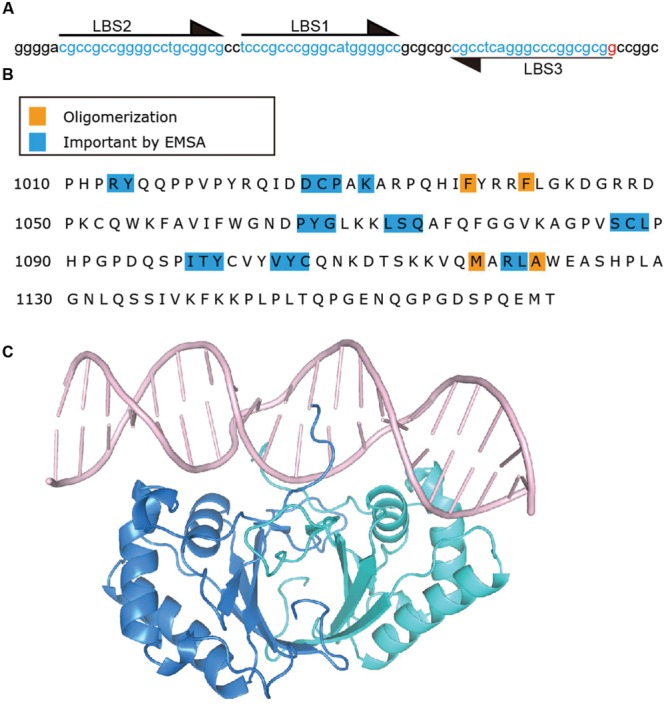
**Latency-associated nuclear antigen binds specific sequence in KSHV TR DNA. (A)** TR sequence with LBS sequences indicated by arrows and highlighting. **(B)** C-terminal LANA residues. Residues which impact DNA binding by EMSA (Kelley-Clarke et al., 2007b; Han et al., 2010) are highlighted in blue and those involved in oligomerization (Correia et al., 2013; Domsic et al., 2013; Hellert et al., 2013, 2015; Ponnusamy et al., 2015) of LANA dimers are highlighted in orange. **(C)** Crystal structure of C-terminal LANA complexed with LBS1 (Hellert et al., 2015) PDB:4UZB. One LANA monomer is shown in blue, and the other in green.

Latency-associated nuclear antigen binds TR DNA to mediate KSHV DNA replication and to tether episomes to mitotic chromosomes. The finding that LANA acted on KSHV TR associated DNA to mediate episome persistence in the absence of other virus genes led to the hypothesis that LANA can directly interact with TR DNA. Subsequently, a 20 bp sequence in the TR was identified as a LANA binding site (LBS1) and was bound by the LANA C-terminal domain, also referred to as the DNA binding domain (DBD) (Ballestas and Kaye, 2001; Cotter et al., 2001; Garber et al., 2001). Mutagenesis of the LANA binding site revealed that the _6_CCC_8_ nucleotides of LBS1 are critical for LANA interaction. Substitutions of these nucleotides severely impacted LANA binding. In contrast, mutations within other nucleotides had only modest effects on binding (Srinivasan et al., 2004). A second LBS (LBS2) was identified downstream of LBS1 through electrophoretic mobility shift assay (EMSA) and DNase I footprinting experiments (Garber et al., 2002). Compared to LBS1, LBS2 is a low affinity LANA binding site. However, since LBS2 is adjacent to LBS1, LBS1 is capable of facilitating cooperative binding of LANA to LBS2 (Garber et al., 2002). Recently, a third LBS (LBS3) was identified, also adjacent to LBS1, but with inverse orientation compared to LBS1 and LBS2 (Hellert et al., 2015) (**Figure 4A**). Similar to LBS2, LBS3 is a low affinity binding site with about a 100-fold lower LANA binding affinity compared to LBS1. Interestingly, LANA DBD binding to LBS1 and LBS3, or to LBS2–LBS1–LBS3 proceeds in a biphasic manner, with LANA binding to LBS1 (or cooperatively to LBS2–LBS1) in the first phase, and then binding LBS3 in the second phase (Ponnusamy et al., 2015). The biphasic binding to LBS3 could be due to the slightly greater distance between LBS1 and LBS3 as compared to LBS1 and LBS2 (Ponnusamy et al., 2015). LBS3 was previously recognized as a 32 bp sequence essential for LANA mediated replication of TR DNA, and had been termed the replication element (Hu and Renne, 2005). Although all three LBSs share homologous sequence, both LBS2 and LBS3 harbor a cytosine to guanine transversion at position 8 (Hellert et al., 2015). Considering the critical role of this cytosine for LBS1 binding affinity (Srinivasan et al., 2004), it is very likely that the low binding affinities for LANA of LBS2 and LBS3 arises from this cytosine to guanine substitution at position 8. The spacing of 22 bp between each LBS results in location of the LBS’s on the same face of DNA, thereby allowing adjacent LANA dimers to bind cooperatively (Garber et al., 2002; Hu and Renne, 2005; Hellert et al., 2015). In addition to binding viral DNA, LANA is capable of binding host genomic DNA. Chip-seq data shows that host LANA-binding sites are generally found within transcriptionally active promoters with sequence homology to the characterized LBS’s within TR DNA (Lu et al., 2012; Hu et al., 2014; Mercier et al., 2014). A novel LANA binding motif (TCCAT)_3_, which is only present in host LANA-binding sites, and to which LANA binds with low affinity, similar to LBS2, was also confirmed by gel shift analysis (Hu et al., 2014).

Mutagenesis has also defined residues within the LANA DBD as important for DNA binding (**Figure 4B**). LANA mutants which are deficient for, or lack DNA binding ability, are also deficient or abolished for the ability to mediate KSHV DNA replication or episome persistence (Komatsu et al., 2004; Han et al., 2010). The inability of LANA abolished for DNA binding to mediate episome persistence is due both to an inability to replicate KSHV DNA as well as loss of the ability to tether KSHV episomes to mitotic chromosomes to segregate episomes to daughter nuclei. C-terminal LANA also mediates LANA’s self-association, and self-association is necessary for DNA binding; deletions in C-terminal LANA that result in the loss of LANA self-association also result in loss of DNA binding and episome persistence (Schwam et al., 2000; Komatsu et al., 2004). As noted above, C-terminal LANA concentrates to percicentromeres and peri-telomeres of a subset of mitotic chromosomes in addition to binding KSHV TR DNA. Alanine scanning mutagenesis of C-terminal LANA revealed that different subsets of residues are important for binding to TR DNA versus mitotic chromosomes (Kelley-Clarke et al., 2007b; Han et al., 2010). Therefore, C-terminal LANA does not appear to attach to mitotic chromosomes through recognition of its TR DNA binding sequence.

#### Structure of the LANA DNA Binding Domain

The x-ray crystal structures of the KSHV and MHV68 LANA DNA binding domains were recently solved (Correia et al., 2013; Domsic et al., 2013; Hellert et al., 2013, 2015; Ponnusamy et al., 2015). These revealed an alpha-beta fold that assembles as a dimer, and that is structurally similar to the EBV EBNA1 and papillomavirus E2 DBDs. The hydrophobic interface of LANA DBD dimerization interface is mediated by an eight-strand anti-parallel intermolecular β-barrel to which each monomer contributes four β-sheets (Domsic et al., 2013; Hellert et al., 2013, 2015). However, in contrast to EBNA1 and E2, a unique feature of LANA is a positive electrostatic patch on the surface opposite of the DNA binding surface. This positive patch serves to interact with BET proteins, although BET proteins also interact with a second region (residues 1125–1129) in C-terminal LANA (Hellert et al., 2013). Mutation of the positive patch reduced LANA’s ability to mediate DNA replication and episome persistence, and mutation of the peripheral region of the positive patch generated the most severe effects, compared to mutation of other portions of the patch (Correia et al., 2013; Domsic et al., 2013; Hellert et al., 2013; Li et al., 2015). These deficiencies in replication and maintenance were unrelated to BET protein binding (Correia et al., 2013; Li et al., 2015). Mutation of the analogous positive patch in MHV68 LANA impacted the ability of MHV68 to establish latent infection in mice (Correia et al., 2013; Hellert et al., 2013).

**FIGURE 5:**
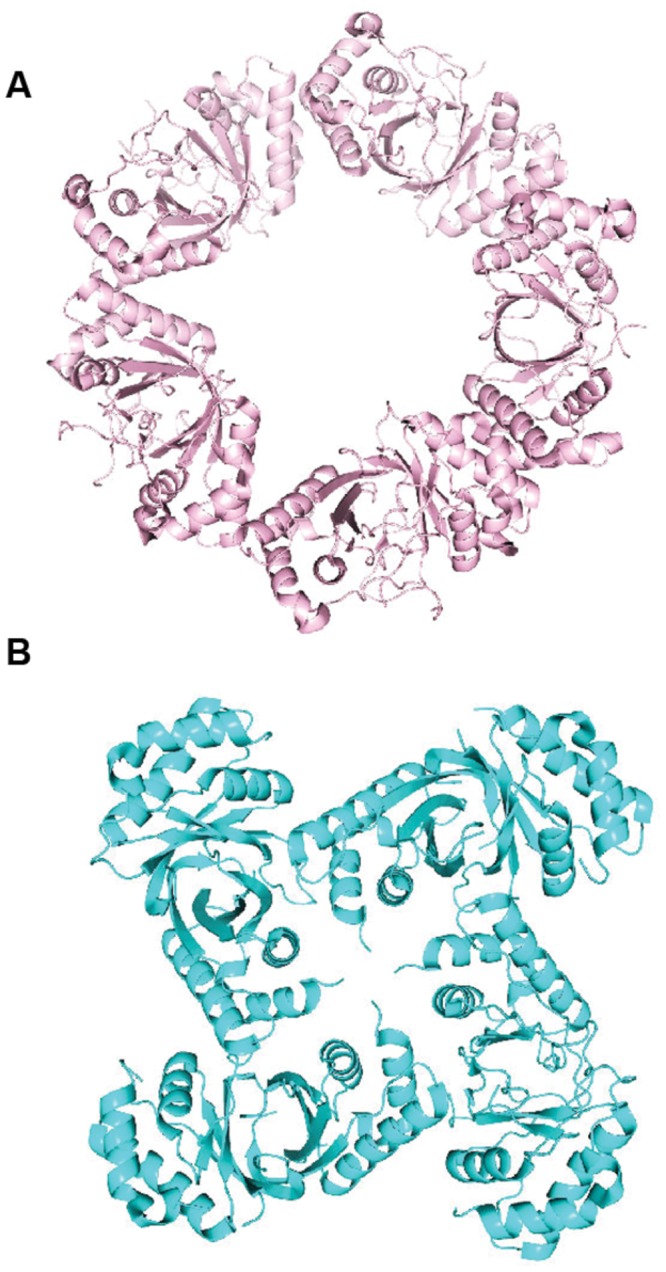
**Higher-order crystal structures of the KSHV LANA DNA binding domain. (A)** Ring form structure of the KSHV LANA DNA binding domain composed of 5 dimers (from PDB:4K2J.) (Domsic et al., 2013). **(B)** Non-ring form tetramer structure of KSHV LANA DNA binding domain (from PDB:5A76.) (Ponnusamy et al., 2015).

Another notable finding of the LANA structure was an oligomerization interface between LANA dimers. This ability of adjacent LANA dimers to interact underlies LANA’s cooperative binding to adjacent DNA binding sites. Disruption of this interface results in loss of LANA cooperative binding to LANA binding sites such as LBS1 and LBS2, deficient DNA replication, and deficient episome persistence (Domsic et al., 2013; Hellert et al., 2013). Importantly, the hydrophobic interface that mediates the dimer–dimer interaction results in a bend and also is a flexible pivot point that allows rotation and bending between two adjacent dimers (Ponnusamy et al., 2015). Variation in the degree of bend between adjacent dimers can result in LANA DBD dimers oligomerizing to form ring structures consisting of different numbers of dimers (e.g., four or five dimers) (**Figure 5A**). In these LANA structures, the DNA binding surface of each dimer is on the exterior face (Domsic et al., 2013; Hellert et al., 2013, 2015). In addition, LANA dimers can form non-ring structures (**Figure 5B**) as well as a spiral (Hellert et al., 2015; Ponnusamy et al., 2015). MHV68 LANA also forms oligomers through interactions between adjacent dimers, which mediates cooperative binding to adjacent binding sites in MHV68 TR DNA. However, in contrast to KSHV LANA, MHV68 LANA forms a rigid, linear conformation compared to KSHV LANA’s bent, flexible configuration (Hellert et al., 2013; Ponnusamy et al., 2015).

#### LANA Recognition of DNA

A recent x-ray crystal structure of the KSHV LANA DBD bound to the high affinity LBS1 recognition site was solved, revealing insight into LANA’s recognition of DNA (Hellert et al., 2015) (**Figure 4C**). To obtain the structure, point mutations were introduced in the DBD in order to obtain well-behaved complexes with DNA. Notably, and in contrast to EBNA1 or E2’s recognition of DNA (Hegde et al., 1992; Bochkarev et al., 1996), the LANA DNA dimer binds LBS1 with remarkable asymmetry, with the primary sequence recognition occurring within G5 to G10 of the 20 bp LBS1. These findings are consistent with mutagenesis studies of LBS1 as discussed above, which revealed that alteration of LBS1 _6_CCC_8_ nucleotides resulted in severe deficiency of LANA binding as compared to other mutations (Srinivasan et al., 2004). The N-terminal arm residues of one monomer, and helices 1 and 2 of the second monomer make sequence specific contacts with base pairs G5 to G10. The corresponding residues of the other monomer within the dimer make contact only with the DNA backbone. Deletion (LANAΔ1007–1021) or point mutations (R1013, Y1014) within the N-terminal arm of the LANA DBD ablates TR DNA binding, replication and episome maintenance (Komatsu et al., 2004; Domsic et al., 2013). A bend of 25° is introduced in DNA by the bound LBS1 DNA, which is less than the previously reported bend of 57°, but could be due to crystal packing constraints (Wong and Wilson, 2005).

In addition to sequence specific binding, the LANA DBD was also proposed to bind DNA in a sequence independent manner (Hellert et al., 2015). In both the ring forms (**Figure 5A**) and spiral structures (as described above) formed by oligomerizing LANA dimers, the sequence specific binding surface is located on the outside of the rings or spiral, and the positive electrostatic patch is located on the inside of the rings or circle, to form a continuous, positively charged inner surface. The inner diameter of the rings or spiral is large enough to accommodate double stranded DNA. In fact, negative staining electron microscopy demonstrated that LANA DBDs mutated for sequence specific binding, were able to form complexes on an arbitrary dsDNA fragment. Mutations of the oligomerization surface or of the positive electrostatic patch abolished the LANA DBD’s ability to complex with the DNA fragment (Hellert et al., 2015). It is possible that related, higher order structures of LANA may account for the LANA dots observed when LANA concentrates at episomes in cells.

### Role of Internal LANA Sequences in Viral Persistence

Although both the N-terminal LANA chromosome attachment region and the C-terminal DBD are essential for episome maintenance, they are insufficient for efficient KSHV episome persistence. Accordingly, fusion of N-terminal (aa 1–32) and C-terminal (aa 929–1162) LANA is highly deficient for episome maintenance, despite its ability to bind mitotic chromosomes and TR DNA at WT levels (De Leon Vazquez and Kaye, 2011). The internal LANA sequence can be divided into two major components: unique sequence, located between amino acids 33 and 331, which includes a proline-rich region from amino acids 63 to 271, and large region of imperfect repeat elements between residues 332 and 931 (**Figure 1**). Both these regions contribute to viral episome maintenance.

The unique internal sequence exerts an important role for episome maintenance. LANAΔ33–273, which contains a deletion of amino acids 33 to 273, was highly impaired for episome maintenance of a plasmid containing eight TR elements. LANAΔ33–273 was also deficient for the ability to replicate TR DNA in transient assays. A number of different host cell proteins involved in DNA replication have been described to interact physically or functionally with LANA including origin recognition complex (ORC) proteins 1-6, mini-chromosome maintenance complex (MCM), HBO1, a histone acetyltransferase important for DNA replication licensing, topoisomerase IIbeta (TopoIIbeta), ubiquitin specific protease USP7, structure-specific recognition protein 1 (SSRP1), replication proteins A1 and A2, replication factor C (RFC), and the proliferating cell nuclear antigen (PCNA) (Lim et al., 2002; Stedman et al., 2004; Verma et al., 2006; Jager et al., 2012; Purushothaman et al., 2012; Shamay et al., 2012; Sun Q. et al., 2014, Sun et al., 2015). However, none of these proteins have yet been mapped to this region of LANA, although deletion of residues 262–320, which partially overlaps residues 33–273, abolished RFC interaction. It is possible that one of these, or other host cell proteins may interact with this region to exert effects on LANA’s DNA replication.

In addition to replication deficiency, the unique internal sequence 33–331 was found to be important for segregation of TR DNA to progeny nuclei. Segregation ability was assessed with a GFP reporter plasmid containing two TR elements that are incompetent for LANA mediated DNA replication due to deletion of LBS3 (formerly termed the replication element) (Hu and Renne, 2005; Skalsky et al., 2007). LANA still maintains the ability to bind this plasmid since LBS1 and LBS2 remain present. Despite preservation of DNA binding and chromosome binding, which allow tethering of TR DNA to mitotic chromosomes, LANA deleted for residues within 33–331 was deficient for the ability to segregate DNA to daughter nuclei (as assessed by retention of GFP expression), while deletion mutants containing residues 33–331 were WT for segregation (De Leon Vazquez et al., 2013). It is possible that this sequence may interact with a host cell protein important for a process such as ensuring that histones bound with LANA are deposited onto newly formed nucleosomes during DNA replication. Alternatively, this region could be responsible for ensuring that LANA molecules with bound episomes are distributed onto sister chromatids. It is possible that facilitating chromatin transcription (FACT), which disrupts and reassembles nucleosomes through removal and deposition of histones H2A/H2B, could have such a role since LANA interacts with the FACT component, SSRP1 (Reinberg and Sims, 2006; Tan et al., 2006; Hu et al., 2009). It is also possible that HMGA1 or HMGB1, which interact with LANA and remodel chromatin (Shamay et al., 2012), could have a role in LANA segregation. The interactions of HMGA1, HMGB1, and SSRP1 within LANA have not been mapped. The LANA interacting kinetochore proteins, CENPF or Bub1, are other intriguing possibilities, and these interact with LANA 1–340 and amino acids 842–1162, which therefore includes the unique internal region (Xiao et al., 2010).

Latency-associated nuclear antigen also has several motifs within the unique internal sequence that could have roles in replication or segregation. A bipartite LANA SUMO-interacting motif (SIM), which interacts with SUMO-2 modified proteins, such as the transcription factor KAP1, is located at residues 244–250 and 264–270, and has been shown to be important for LANA mediated episome persistence (Campbell and Izumiya, 2012; Cai et al., 2013). LANA contains at least one phosphorylation site within amino acids 31–52, and is also phosphorylated at additional sites within 91–340 as discussed above (Fujimuro et al., 2005; Liu et al., 2007; Cha et al., 2010). In addition, there is a suppressors of cytokine signaling (SOCS) box-like motif which contains an Elongin B and C box located at LANA residues 212–222. LANA also has another component of the SOCS box motif, a Cullin box, which is spatially separated from the elongin B/C motif, and is located at residues 1085–1100 within the C-terminal domain (Cai et al., 2006). It is possible that any of these sites within the unique internal sequence may contribute to LANA mediated segregation or replication.

The LANA internal repeat region consists of glutamine rich and acidic imperfect repeat elements spanning from amino acids 332–931 (Russo et al., 1996). The length of each region and the number of repeats varies depending on the virus isolate (Rainbow et al., 1997). Deletion of the entire repeat region between residues 332 to 929 diminishes LANA’s ability to mediate episome persistence of a small plasmid containing eight TRs to a modest degree compared to deletion of most of the unique internal sequence (residues 33–273) (~10-fold vs. ~275-fold, respectively) (De Leon Vazquez et al., 2013). However, deletion of the repeat elements had a potent effect on persistence of the entire KSHV genome. A BAC containing the KSHV genome with LANA deleted for amino acids 329–931 lost the ability to persist. After transfection of cells with this BAC, no colonies formed under drug selection. This loss was not due to a major replication defect, as the LANA mutant retained the ability to replicate a plasmid containing TR DNA in a transient assay (Alkharsah and Schulz, 2012). Therefore, both the unique internal sequence and the internal repeat elements contribute to LANA episome persistence function.

### KSHV Episome Persistence Is a Multi Step Process and May Require Epigenetic Changes for Longterm Stability

**FIGURE 6:**
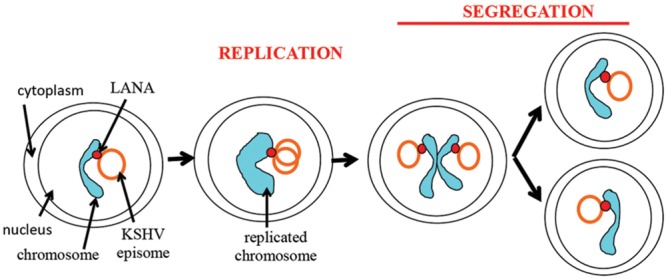
**Schematic representation of episome persistence through the cell cycle.** LANA tethers the episome to the cellular chromosome. During S phase, the viral episome replicates once in concert with cell DNA (Verma et al., 2007). A potential model is shown of two replicated episomes attaching to adjacent sister chromatids and segregating to daughter nuclei through tethering to chromatids.

As discussed above, there are two steps necessary for viral episome maintenance in proliferating cells (**Figure 6**). First, the viral episomes replicate in concert with cell DNA once per cell cycle using a semiconservative mode of replication (Verma et al., 2007). Second, replicated episomes must be efficiently dispatched to daughter cells. This critical process involves tethering the viral genomes to cellular mitotic chromosomes to allow migration of episomes to the nuclei of daughter cells, thereby avoiding viral DNA degradation in the cytoplasm. Although the terms “segregation” or “partitioning” are sometimes used to describe different components of this process, such terms tend to be arbitrary as the process is a continuous one that accomplishes the global goal of ensuring that replicated episomes are distributed to daughter nuclei.

The attachment of LANA bound KSHV episomes to nucleosomes is likely a multi-step process. For instance, histones are removed from DNA prior to its replication and re-deposited following replication. It is possible that LANA may remain bound to histones throughout this process or that LANA may be removed from histones, and then reattached to nucleosomes following DNA replication. Further, if LANA deposition on sister chromatids is not random, a mechanism must exist to ensure equal episome distribution to each sister chromatid.

A faithful segregation system of newly replicated episomes to daughter cells would seemingly be the most efficient method of distribution. Notably, it has been observed that a similar KSHV genome copy number appears to be maintained in infected cells within a population (Ueda et al., 2006), consistent with a faithful partitioning mechanism. In addition, equivalent amounts of chromosome associated GFP LANA appeared to partition in dividing, uninfected cells, also suggesting faithful partitioning (Tetsuka et al., 2004). However, work using a KSHV BAC marked with tandem lactose operator sequences to allow binding of a lactose repressor protein fused to fluorescent protein suggested KSHV may partition randomly (Norby et al., 2012). For EBV, episome segregation has been shown to be a non-random process (Nanbo et al., 2007).

Despite the persistence of stable copy numbers of KSHV episomes in infected cell lines, such as primary effusion lymphoma (PEL) cells, the establishment of stable KSHV persistence is a relatively inefficient process *in vitro* (Grundhoff and Ganem, 2004; Ueda et al., 2006). In fact, the use of a selection marker is necessary for efficient selection of cells with persistent maintenance of episomes (Tetsuka et al., 2004; Ueda et al., 2006). This situation is similar to that of EBV, where the establishment of stable EBNA1 mediated episome persistence of oriP plasmids is also inefficient (Leight and Sugden, 2001). It is likely that *cis* acting, epigenetic changes in the viral episome are necessary for stable persistence, and that such changes occur at a relatively low frequency. However, once they have occurred, episome maintenance remains stable (Grundhoff and Ganem, 2004; Skalsky et al., 2007). It has been hypothesized that lytic infection may be important for KS tumorigenesis due to the inefficiency of KSHV persistence following infection. Lytic infection could potentially recruit new infected cells to replace those that lose KSHV infection (Grundhoff and Ganem, 2004). However, an alternative explanation is that the local microenvironment *in vivo* may provide critical conditions that are necessary for efficient persistence of KSHV following infection.

## Conclusion

**FIGURE 7:**
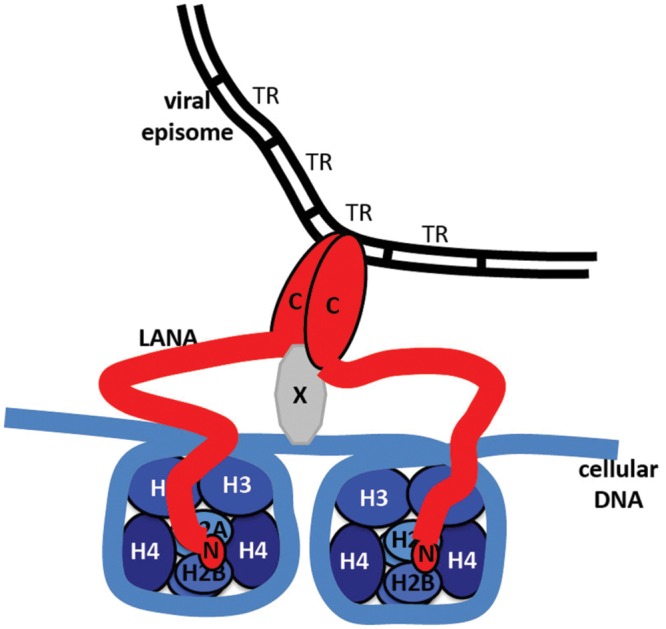
**Model of LANA tethering the KSHV genome to a chromosome.** N-terminal LANA (N) binds to core histones H2A/H2B. C-terminal LANA (C) self-associates, binds to KSHV TR DNA in the KSHV genome, and binds to a putative protein (X) that associates with the chromosome.

Kaposi’s sarcoma-associated herpesvirus genome persistence is mediated by the viral LANA protein. LANA mediates viral DNA replication and tethers KSHV DNA (**Figure 7**) to mitotic chromosomes to segregate episomes to progeny nuclei (**Figure 6**). To tether episomes to chromosomes, N-terminal LANA directly interacts with the folded portion of the nucleosome at the conserved acidic patch at the interface of histones H2A and H2B. C-terminal LANA also interacts with chromosomes, likely by binding a chromosomal protein. In addition, C-terminal LANA mediates the interaction with viral DNA by binding sequence within the TR elements. The structure of the LANA DBD was recently solved and suggests that cooperative binding of LANA to its adjacent binding sites within each TR is mediated by interactions between LANA dimers. LANA dimers can assemble into higher-order structures, mediated by dimer-dimer interactions. LANA interacts with a number of cellular proteins, including at the chromosome. Through these interactions, LANA exerts its functions, including KSHV DNA replication and segregation. Although N and C-terminal LANA are necessary, they are not sufficient for efficient episome maintenance. LANA internal regions also exert important roles necessary for maintenance of episomes. The essential role of LANA in episome persistence makes it an ideal target for inhibition for the treatment and prevention of KSHV malignancies. Inhibition of any critical component of LANA’s episome persistence function, including binding to TR DNA or to mitotic chromosomes, is expected to abolish KSHV persistence, and eliminate virus infection from proliferating cells.

## Author Contributions

All authors have made substantial, direct and intellectual contribution to the work, and approved it for publication.

## Conflict of Interest Statement

The authors declare that the research was conducted in the absence of any commercial or financial relationships that could be construed as a potential conflict of interest.
